# Dexamethasone and insulin activate serum and glucocorticoid‐inducible kinase 1 (SGK1) via different molecular mechanisms in cortical collecting duct cells

**DOI:** 10.14814/phy2.12792

**Published:** 2016-05-24

**Authors:** Morag K. Mansley, Gordon B. Watt, Sarah L. Francis, David J. Walker, Stephen C. Land, Matthew A. Bailey, Stuart M. Wilson

**Affiliations:** ^1^ Division of PharmacySchool of Medicine, Pharmacy and HealthDurham University Queen's CampusStockton‐on‐TeesUK; ^2^ Medical Research InstituteCollege of Medicine, Dentistry and NursingNinewells Hospital and Medical SchoolUniversity of DundeeDundeeUK; ^3^ The British Heart Foundation Centre for Cardiovascular ScienceQueen's Medical Research InstituteUniversity of EdinburghEdinburghUK

**Keywords:** Cortical collecting duct, epithelial Na^+^ channel, Na^+^ transport, renal Na^+^ retention, Ussing chamber

## Abstract

Serum and glucocorticoid‐inducible kinase 1 (SGK1) is a protein kinase that contributes to the hormonal control of renal Na^+^ retention by regulating the abundance of epithelial Na^+^ channels (ENaC) at the apical surface of the principal cells of the cortical collecting duct (CCD). Although glucocorticoids and insulin stimulate Na^+^ transport by activating SGK1, the responses follow different time courses suggesting that these hormones act by different mechanisms. We therefore explored the signaling pathways that allow dexamethasone and insulin to stimulate Na^+^ transport in mouse CCD cells (mpkCCD_cl4_). Dexamethasone evoked a progressive augmentation of electrogenic Na^+^ transport that became apparent after ~45 min latency and was associated with increases in SGK1 activity and abundance and with increased expression of SGK1 mRNA. Although the catalytic activity of SGK1 is maintained by phosphatidylinositol‐OH‐3‐kinase (PI3K), dexamethasone had no effect upon PI3K activity. Insulin also stimulated Na^+^ transport but this response occurred with no discernible latency. Moreover, although insulin also activated SGK1, it had no effect upon SGK1 protein or mRNA abundance. Insulin did, however, evoke a clear increase in cellular PI3K activity. Our data are consistent with earlier work, which shows that glucocorticoids regulate Na^+^ retention by inducing *sgk1* gene expression, and also establish that this occurs independently of increased PI3K activity. Insulin, on the other hand, stimulates Na^+^ transport via a mechanism independent of *sgk1* gene expression that involves PI3K activation. Although both hormones act via SGK1, our data show that they activate this kinase by distinct physiological mechanisms.

## Introduction

Within the kidneys, ~99% of filtered Na^+^ is reabsorbed via ion transport processes within the epithelia of the renal tubules and, while most of this reabsorption occurs via constitutively active Na^+^ transport systems in the proximal nephron, the amount of Na^+^ recovered within the distal nephron is under hormonal control. This regulated process is therefore central to whole body sodium and water homeostasis and the long‐term regulation of blood pressure (Blazer‐Yost et al. 1998; De La Rosa et al. 2002; Loffing and Korbmacher 2009). Na^+^ retention within this nephron segment is primarily controlled via the renin–angiotensin–aldosterone system (RAAS), which becomes active under conditions of hypovolemia and hyponatremia. The final effector in this system is aldosterone, a steroid hormone that promotes Na^+^ retention via the epithelial Na^+^ channel (ENaC) by binding to cytoplasmic mineralocorticoid receptors in the principal cells of the cortical collecting duct. Many drugs used to treat hypertension (angiotensin‐converting enzyme inhibitors, mineralocorticoid receptor antagonists) lower blood pressure by disrupting the RAAS and thus promoting diuresis/natriuresis. However, several other hormones contribute to the control of Na^+^ transport in this nephron segment (Loffing and Korbmacher 2009) and a body of work suggests that inappropriate stimulation of ENaC by glucocorticoids may contribute to the hypertension seen in conditions such as diabetes, obesity, and metabolic syndrome that are characterized by glucocorticoid excess (Bailey et al. 2008, 2009; Hunter et al. 2014). Moreover, as well as its role in carbohydrate metabolism, insulin can also activate ENaC in the distal nephron (Blazer‐Yost et al. 1998; Loffing and Korbmacher 2009) and, while this effect normally has little significance, it may contribute to the hypertension and edema that can develop in patients with type II diabetes who receive insulin in combination with drugs that sensitize cells to this hormone (see, e.g., Guan et al. 2005; Loffing and Korbmacher 2009; Wilson et al. 2010). It is therefore important to understand the mechanisms that allow glucocorticoids and insulin to regulate Na^+^ retention within the distal nephron, and earlier work indicates that both hormones act via the serum and glucocorticoid‐inducible kinase 1 (SGK1), a protein kinase that contributes to the control of Na^+^ retention by regulating the abundance of ENaC at the cell surface (De La Rosa et al. 1999; Kobayashi and Cohen 1999; Park et al. 1999; Wang et al. 2001; Faletti et al. 2002; Loffing and Korbmacher 2009). However, despite this similarity, it is also clear that the responses to dexamethasone, a synthetic glucocorticoid, and insulin follow different time courses (see, e.g., Mansley and Wilson 2010a, b) and this discrepancy suggests that these hormones may act via different mechanisms. This study therefore compares the effects of these hormones upon the activity/abundance of SGK1 in mouse cortical collecting duct cells. By combining these methods with electrometric assays of ENaC activity we have defined the mechanisms that allow these hormones to control ENaC‐mediated Na^+^ transport. While dexamethasone and insulin both increase SGK1 activity, these hormones control this regulatory kinase via physiologically distinct signaling pathways that can function independently of each other.

## Materials and methods

Since the methods used are described elsewhere (Bens et al. 1999; Mansley and Wilson 2010b; Scott et al. 2010) only brief details are presented here. Experiments were undertaken using mpkCCD_cl4_ murine cortical collecting duct cells grown to confluence on Costar Transwell or Snapwell membranes (Corning, Flintshire, UK) and deprived of hormones/growth factors for ~48 h (Bens et al. 1999; Mansley and Wilson 2010b). Amiloride, dexamethasone, insulin, cyclohexamide, and general laboratory chemicals were from Sigma (Poole, Dorset, UK). Antibodies against the Thr^346/356/366^‐phosphorylated and total forms of the protein encoded by n‐myc downstream‐regulated gene 1 (NDRG1) and the antibody against SGK1 were provided by the MRC Protein Phosphorylation/Ubiquitinylation Unit (University of Dundee, Prof. D.R. Alessi). The antibodies against the Ser^473^‐phosphorylated, Thr^308^‐phosphorylated and total forms of protein kinase B (PKB, also known as Akt) were from Cell Signalling (Hertfordshire, UK).

The electrometric properties of cells on Transwell membranes were assessed using an EVOM^2^ epithelial volt‐ohm‐meter (WPI, Hertfordshire, UK) to measure transepithelial resistance (*R*_t_) and voltage (*V*_t_, relative to earth electrode in the basolateral bath). The transepithelial current needed to hold *V*_t_ at 0 mV (equivalent short circuit current, *I*_Eq_) was then calculated (*I*_Eq_ = *V*_t_/*R*_t_). Cells on Snapwell membranes were mounted in Ussing chambers where *V*_t_ was monitored (Mansley and Wilson 2010b) under open circuit conditions. The changes in *V*_t_ induced by the repeated, brief (20 s) injections of transepithelial current (−10 *μ*A cm^−2^) were also monitored so that *R*_t_ (*R*_t_ = Δ*V*_t_/Δ*I*_t_) and *I*_Eq_ could be determined. Experiments were terminated by adding 10 *μ*mol/L amiloride to the apical bath, and amiloride‐sensitive short circuit current (*I*_Amil_) then calculated. Since 1 *μ*A is defined as 1 *μ*C s^−1^, we quantified electrogenic Na^+^ flux (*J*_Na_) using the expression *J*_Na_ = *I*_Eq_ × 60/*F*, where *F* is the Faraday constant (9.649 × 10^4^, C mol^−1^). Experimentally induced changes in SGK1 activity were monitored by quantifying (Western analysis) the phosphorylation of residues (Thr^346/356/366^) within an endogenous protein (NDRG1) that are phosphorylated by SGK1 but not by other closely related kinases (Murray et al. 2004; Inglis et al. 2009). The activity of phosphatidylinositide‐OH‐3‐kinase (PI3K)/phospholipid‐dependent kinase 1 (PDK1) pathway was assayed by monitoring the phosphorylation of a residue (Thr^308^) within PKB that is phosphorylated in a PI3K‐dependent manner by PDK1. Similarly, the activity of the PI3K/target of rapamycin signaling complex 2 (TORC2) pathway was assessed by monitoring the phosphorylation status of PKB‐Ser^473^ (Alessi et al. 1996; Bayascas and Alessi 2005; Sarbassov et al. 2005). The abundance of mRNA transcripts encoding SGK1 and the ENaC *α* subunit (*α*‐ENaC) were determined by real time, quantitative PCR. RNA was therefore extracted from control/hormone‐stimulated cells grown on Transwell membranes (Promega SV RNA isolation kit) and 1 *μ*g aliquots transcribed into cDNA using Maloney Monkey Leukemia Virus (MMLV) reverse transcriptase. The abundance of each target was then determined using Qiagen Quantitect Primers and a Rotorgene Q H2M qPCR machine (Scott et al. 2010). All experiments were undertaken using paired experimental designs, pooled data are shown as mean ± SEM and values of *n* refer to the number of times a protocol was repeated using cells at different passage. Statistical significances were tested using Student's paired *t*‐test or analysis of variance (ANOVA), with Dunnett's post hoc test, as appropriate.

## Results

### Dexamethasone induced Na^+^ absorption and activation of SGK1

**Figure 1:**
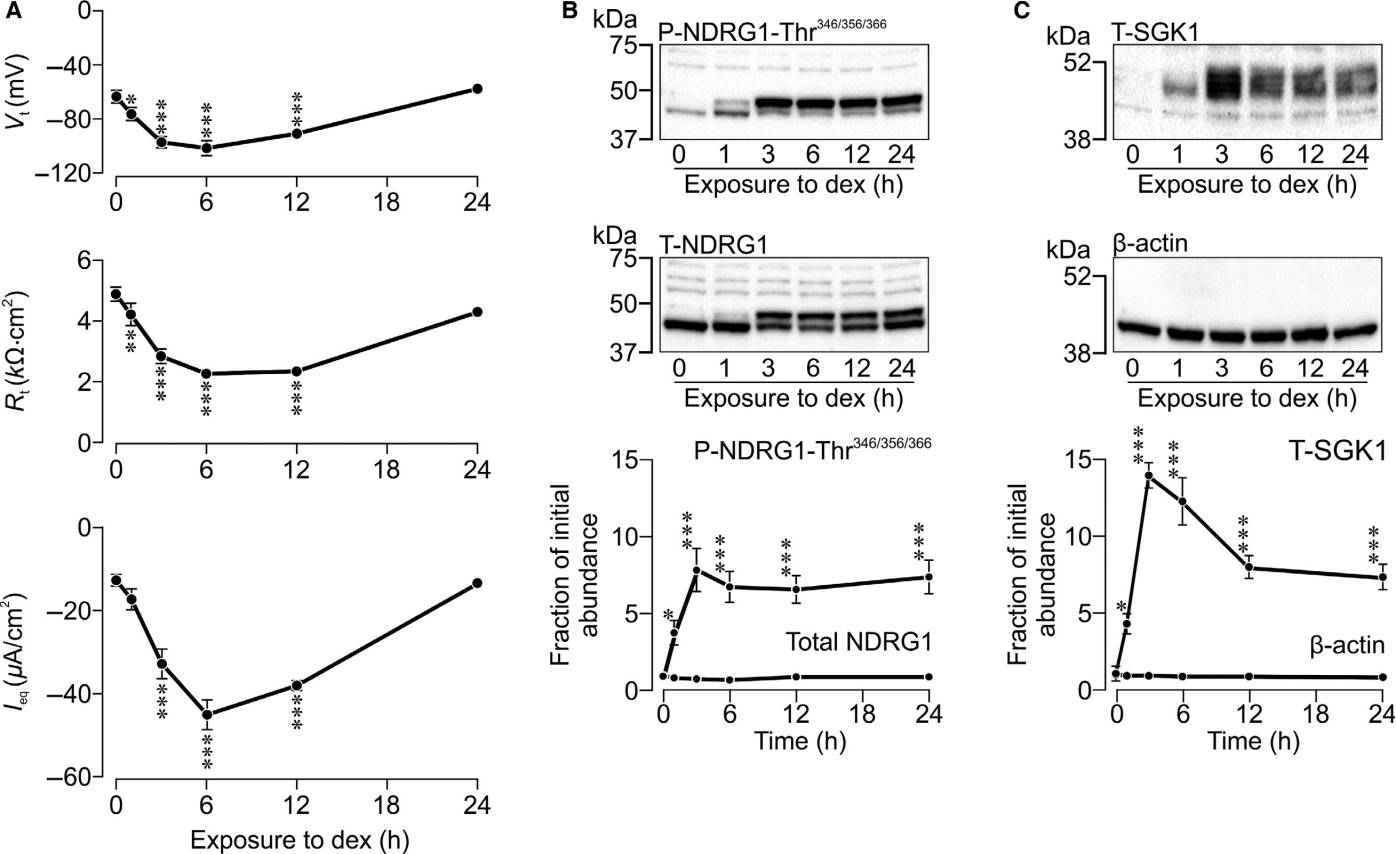
Effects of dexamethasone upon transepithelial ion transport and cellular SGK1 activity/abundance. (A) Transepithelial voltage (*V*_t_) and resistance (*R*_t_) were measured across monolayers of mpkCCD_cl4_ cells grown on Transwell membranes following treatment with dexamethasone (Dex, 0.1 *μ*mol/L) over 0–24 h. *I*_eq_ was subsequently calculated using Ohm's law. Each time point corresponds to a separate monolayer of cells, values shown are mean ± SEM (*n* = 4) compared by one‐way analysis of variance (ANOVA), ***P < 0.001. (B) Effects of dex (0–24 h) on SGK1 activity (B) or abundance (C) determined by phosphorylated NDRG1–Thr^346/356/366^ and total SGK1, respectively, are shown in representative blots. Changes to either total NDRG1 protein (B) or *β*–actin (C) were also monitored. Line graphs show densitometric analysis (*n* = 4) of repeated blots with values shown as mean ± SEM compared by one‐way ANOVA/Dunnett's post hoc test, *P < 0.05, ***P < 0.001.

Studies undertaken using the epithelial volt‐ohm‐meter showed that *V*_t_ and *R*_t_ were normally ~–60 mV and ~5 kΩ∙cm^2^, respectively, and analysis using Ohm's Law established that *I*_Eq_ was ~12 *μ*A cm^−2^ (Fig. 1A). *J*_Na_ (see Methods) was therefore ~7.5 nmol cm^−2^ min^−1^. Since ~95% of the current generated by mpkCCD_cl4_ cells can be blocked by amiloride (Bens et al. 1999; Mansley and Wilson 2010b), these data confirm that these cells absorb Na^+^ via ENaC. Dexamethasone (0.1 *μ*mol/L, 0–24 h), hyperpolarized *V*_t_, and reduced *R*_t_ and these effects became apparent within ~1 h and, at the peak of the response (6 h), the magnitude of *I*_Eq_ was ~4 fold greater than control (Fig. 1A). However, after 24 h, the electrical properties of the cells had returned to their basal values and this response cannot, therefore, be sustained for prolonged periods. Subsequent analysis of protein extracted from these cells showed that dexamethasone increased the abundance of Thr^346/356/366^‐phosphorylated NDRG1 without altering the overall NDRG1 expression level (Fig. 1B). This synthetic hormone thus promotes phosphorylation of NDRG1‐Thr^346/356/366^ (Murray et al. 2004; Inglis et al. 2009). This response became apparent within 1 h, peaked after ~3 h and persisted for at least 24 h. Throughout this study, we have assumed that the increased phosphorylation of NDRG1‐Thr^346/356/366^ provides a biomarker of increased SGK1 activity since these residues are physiological substrates for SGK1 but not for closely related kinases including PKB (Murray et al. 2005) and as studies of genetically modified mice have shown that NDRG1‐Thr^346/356/366^ phosphorylation is abolished by deletion of the *sgk1* gene (Murray et al. 2004). However, despite the clear increase in SGK1 activity, studies using several commercially available antibodies against SGK1 failed to detect any change to the pattern of protein expression consistent with increased abundance of SGK1 (not shown). We therefore undertook experiments using a sheep antibody (S199D, 3^rd^ bleed) against a peptide sequence (KEAAEAFLGFSYAPPTDSFL) corresponding to residues 412 – 431 of human SGK1. This novel antibody was prepared within the MRC protein phosphorylation unit (MRC‐PPU, University of Dundee) and initial studies revealed strong immunoreactivity against heterologously expressed, hemagglutinin‐tagged SGK1 (HA‐SGK1) and much weaker reactivity against overexpressed HA‐SGK2/3 (C.J. Hastie, MRC PPU, personal communication). In our hands this antibody consistently identified a ~48 kDa protein whose expression was strictly dependent upon stimulation with dexamethasone. This change to the pattern of protein expression was confirmed using a second sheep antibody (S062D) against full‐length SGK1 obtained from the same source. Initial studies using this antibody also revealed strong immunoreactivity against heterologously expressed SGK1 and weak reactivity against SGK2 but not SGK3 (C.J. Hastie, MRC PPU, personal communication). While we cannot exclude the possibility that SGK2 may contribute to the immunoreactive band detected in dexamethasone‐stimulated cells, it is important to stress that SGK2 is not usually considered to be hormone‐inducible. We therefore attribute the increased expression of the 48 kDa band to dexamethasone‐induced expression of SGK1. This response followed a time course very similar to the increase in SGK1 activity (Fig. 1C).

### Dexamethasone and insulin have different effects upon SGK1 expression

**Figure 2:**
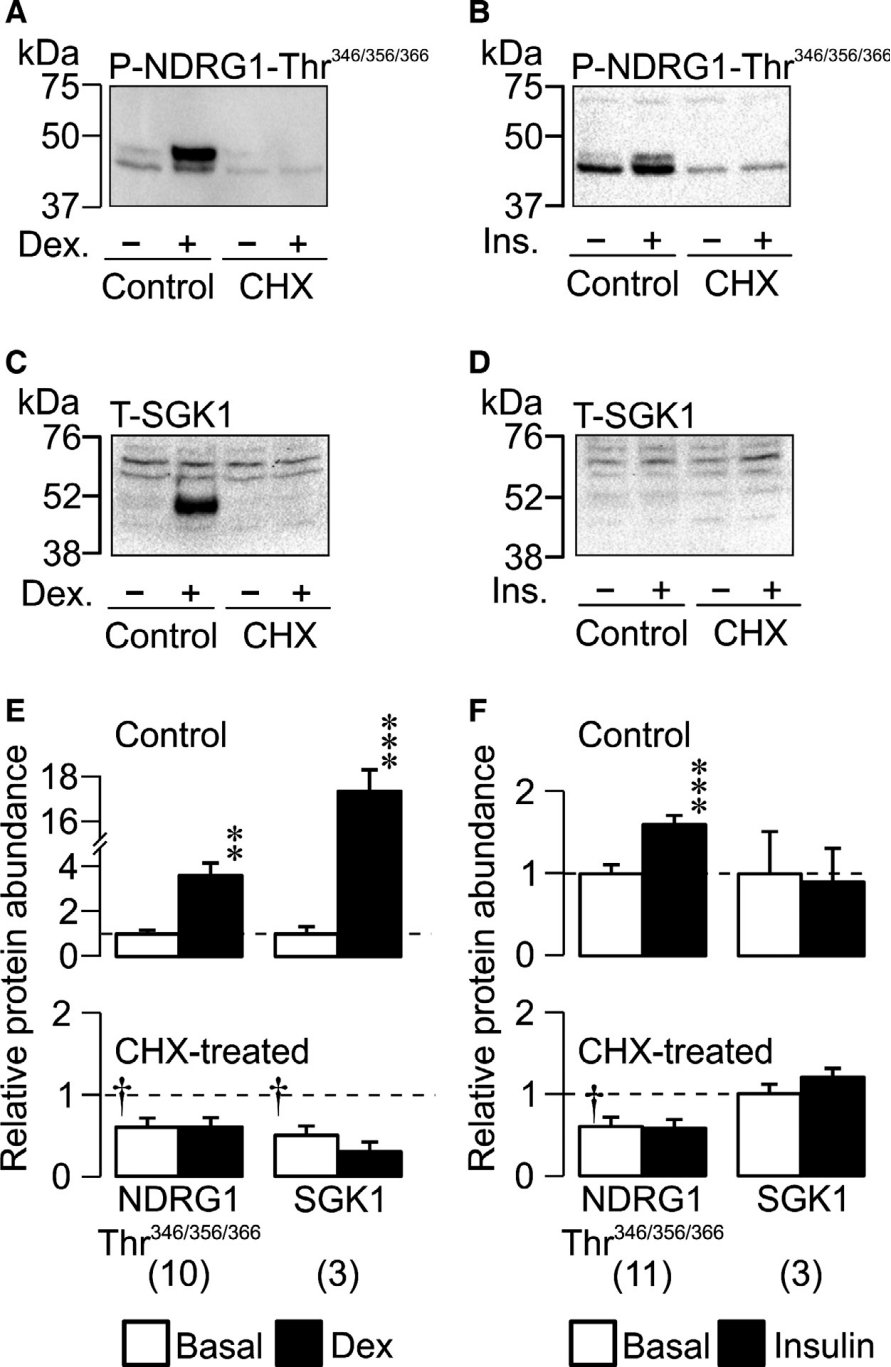
Dexamethasone‐ and insulin‐induced activation of SGK1 is blocked by cyclohexamide. Representative blots of phosphorylated protein from cells pretreated (30 min) with vehicle or 10 *μ*g mL^−1^ cyclohexamide followed by addition of either (A, C, E) dex (100 nmol/L, 3 h) or (B, D, F) insulin (20 nmol/L, 1 h), or respective vehicle control. SGK1 activity and abundance was assessed by monitoring the phosphorylation of NDRG1‐Thr^346,356,366^ (A, B) and total SGK1 (C, D). Mean densitometric analysis of phosphorylated protein pretreated with vehicle control (upper panels) or 10 *μ*g mL^−1^ cyclohexamide (lower panels) followed by treatment with (E) dex (100 nmol/L, 3 h) or (F) insulin (20 nmol/L, 1 h), or respective vehicle control. Values are shown as mean ± SEM,* n* values are denoted in brackets below bars. Statistical significance was calculated using an unpaired t‐test between basal and stimulated conditions in the absence of CHX, **P < 0.01, ***P < 0.001 or between basal conditions in the absence or presence of CHX, ^†^ *P* < 0.05.

**Figure 3:**
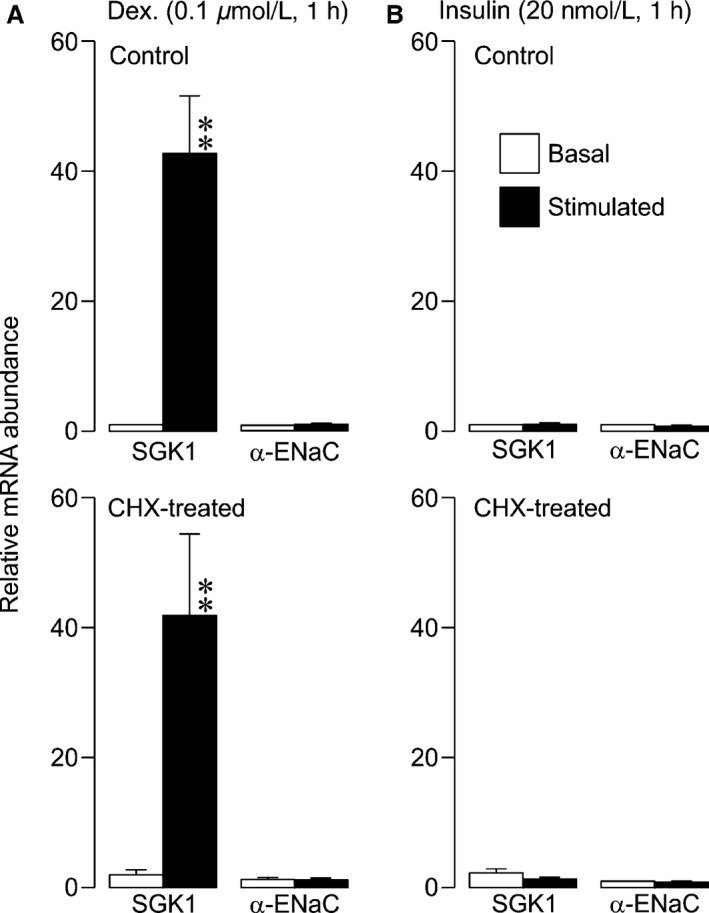
Hormone‐induced changes in mRNA expression. Relative mRNA abundance of SGK1 and *α*–ENaC in mpkCCD_cl4_ cells measured by qRT–PCR of RNA isolated from cells following hormonal treatment. Cells were treated with either (A) dexamethasone (dex, 100 nmol/L) or (B) insulin (20 nmol/L) for 1 h following pretreatment with solvent vehicle (top panels) or 10 *μ*g mL^−1^ cyclohexamide (CHX, lower panels) for 30 min. Values are shown as mean ± SEM, (*n* = 6, dex; *n *= 8, insulin). Statistical significance was calculated using a paired *t*‐test for each target gene investigated between control and treatment group; **P < 0.01.

Further experiments confirmed (Mansley and Wilson 2010a, b) that dexamethasone (Fig. 2A and E) and insulin (Fig. 2B and F) can both activate SGK1 (i.e.*,* promote phosphorylation of NDRG1‐Thr^346/356/366^). However, while dexamethasone also clearly evoked expression of SGK1 protein (i.e., the 48 kDa protein described above Fig. 2C and E) insulin had no effect upon the abundance of this regulatory kinase despite the clear increase in cellular SGK1 activity (Fig. 2D and F). While these data are consistent with the idea (Wang et al. 2001; Itani et al. 2002) that dexamethasone increases cellular SGK1 activity by evoking SGK1 synthesis, they also show that insulin must act via a different mechanism. We therefore used qRT–PCR (see Methods) to explore the effects of these hormones upon *sgk1* gene expression. Dexamethasone caused an unambiguous increase (~40 fold) in the abundance of SGK1 mRNA, but had no effect upon the abundance of mRNA encoding *α*‐ENaC (Fig. 3A). This was initially surprising since *α*‐ENaC is an important part of the Na^+^ transport mechanism whose expression is regulated by steroid hormones (see Sayegh et al. 1999; McTavish et al. 2009). However, earlier work shows that glucocorticoid‐induced *α*‐ENaC gene transcription is a relatively slow response and that significant increases in mRNA abundance are not seen after only 1 h (May et al. 1997). Our data are therefore consistent with the idea that the initiation of glucocorticoid induced Na^+^ transport is dependent upon *sgk1* gene expression (Wang et al. 2001; Faletti et al. 2002). Insulin, on the other hand, had no discernible effect upon the abundance of mRNA encoding SGK1 or *α*‐ENaC (Fig. 3B) and this hormone must therefore increase cellular SGK1 cellular activity via a mechanism independent of *sgk1* gene expression.

### Effects of cyclohexamide on SGK1 expression

Exposing hormone‐deprived cells to cyclohexamide (10 *μ*g mL^−1^, 30 min pre incubation), a substance that abolishes protein synthesis in mammalian cells, reduced (~50%) the abundance of Thr^346/356/366^‐phosphorylated NDRG1 (Fig. 2A and B) without altering the overall NDRG1 expression level (data not shown), a result which indicates loss of cellular SGK1 activity. Although cyclohexamide also seemed to reduce the abundance of SGK1 itself (Fig. 2C and D), this result is associated with considerable uncertainty since SGK1 was barely detectible in hormone‐deprived cells making it difficult to quantify changes in basal expression. While cyclohexamide did abolish the effects of dexamethasone upon the activity and abundance (Fig. 2E) of SGK1, it had no effect upon the dexamethasone‐induced increase in the abundance of SGK1 mRNA (Fig. 3). Cyclohexamide also abolished the effects of insulin upon cellular SGK1 activity (Fig. 2F).

### Dexamethasone and insulin have different effects upon cellular PI3K activity

**Figure 4:**
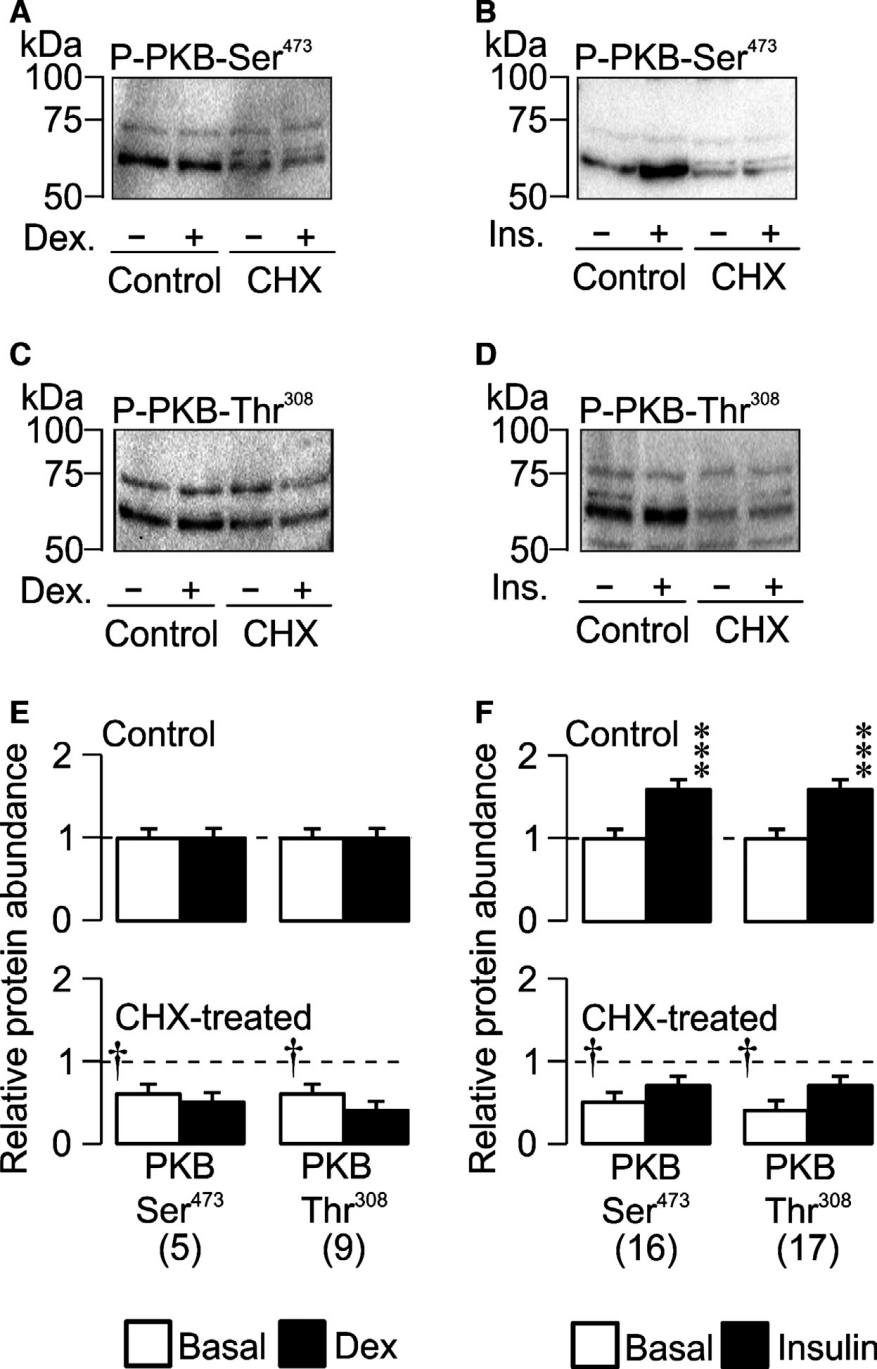
Insulin‐induced activation of PDK1/mTORC2 is blocked by cyclohexamide. Representative blots of phosphorylated protein from cells pretreated (30 min) with vehicle or 10 *μ*g mL^−1^ cyclohexamide followed by addition of either (A, C, E) dex (100 nmol/L, 3 h) or (B, D, F) insulin (20 nmol/L, 1 h), or respective vehicle control. mTORC2 and PDK1 activity were assessed by monitoring the phosphorylation of PKB‐Ser^473^ (A, B) and PKB‐Thr^308^ (C, D). Mean densitometric analysis of phosphorylated protein pretreated with vehicle control (upper panels) or 10 *μ*g mL^−1^ cyclohexamide (lower panels) followed by treatment with (E) dex (100 nmol/L, 3 h) or (F) insulin (20 nmol/L, 1 h), or respective vehicle control. Values are shown as mean ± SEM,* n* values are denoted in brackets below bars. Statistical significance was calculated using an unpaired *t*‐test between basal and stimulated conditions in the absence of CHX, **P < 0.01, ***P < 0.001 or between basal conditions in the absence or presence of CHX, ^†^ *P* < 0.05.

Dexamethasone (0.1 *μ*mol/L, 3 h) did not alter the abundance of the Ser^473^‐ and Thr^308^‐phosphorylated forms of PKB (Fig. 4A,C and E) and had no effect upon the overall PKB expression level (not shown). Since the phosphorylation status of PKB‐Ser^473^/PKB‐Thr^308^ provide biomarkers of cellular PI3K activity (Kobayashi and Cohen 1999; Bayascas and Alessi 2005; García‐Martínez and Alessi 2008), these data show that dexamethasone has no effect upon cellular PI3K activity under the present conditions. Parallel studies of insulin‐stimulated (20 nmol/L, 1 h) cells, on other hand, revealed clear increases in the abundance of Ser^473^‐ and Thr^308^‐phosphorylated PKB (Fig. 4B, D and F) that occurred with no change to the overall PKB abundance (not shown). Insulin thus causes a clear increase in cellular PI3K activity (see Cohen 2006).

### Effects of cyclohexamide on PI3K

Cyclohexamide reduced (~50%) the abundance of the Ser^473^‐ and Thr^308^‐phosphorylated PKB (Fig. 4A–D) without altering the overall PKB expression level (not shown) and also abolished the increased phosphorylation of these residues that is normally seen in insulin‐stimulated cells (Fig. 4B and D). Cyclohexamide therefore reduces basal PI3K activity and abolishes the insulin‐induced activation of this regulatory kinase.

### Effects of cyclohexamide – electrometric responses

**Figure 5:**
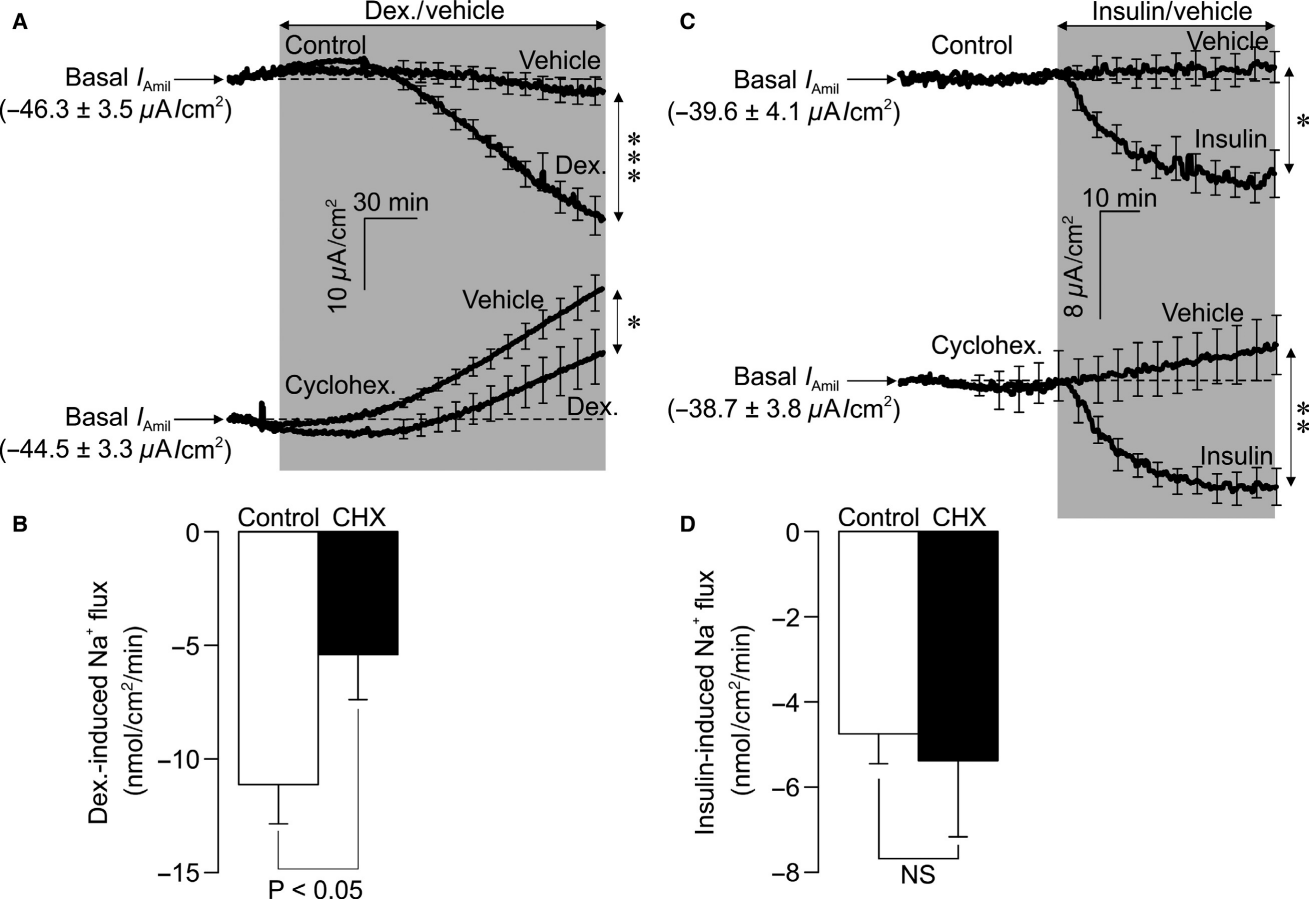
Inhibiting protein translation inhibits stimulation of ENaC by dexamethasone but not insulin. Amiloride‐sensitive *I*_eq_ (*I*_amil_) recorded from cells pretreated (30 min) with vehicle or 10 *μ*g mL^−1^ cyclohexamide followed by addition of either (A) dex (100 nmol/L, 3 h) or (C) insulin (20 nmol/L, 1 h), or their respective vehicle control. Traces are shown 5 min before addition of hormone. Hormonally induced Na^+^ was subsequently calculated and the effects of cyclohexamide on either dex‐induced Na^+^ flux (B) or insulin‐induced Na^+^ flux (D) were compared. Values are shown as mean ± SEM, (*n *= 7 dex; *n* = 6 insulin). Statistical significance was calculated using an unpaired *t*‐test between vehicle and hormone‐treated *I*_amil_ (A,C) or between hormone‐induced Na^+^ flux following control or CHX pretreatment (B, D); *P < 0.05, ***P < 0.001.

Figure 5A show values of *I*_Amil_ measured in confluent cells mounted in Ussing chambers. To analyze such data, the current measured at the onset of each experiment (basal *I*_Amil_) was subtracted from all subsequently recorded data in order to isolate the changes in *I*_Amil_ that occur during each experiment. The recorded current was normally stable (Fig. 5A) and spontaneous changes in *R*_t_ did not occur over the time course of the present experiments. Dexamethasone caused a progressive augmentation of *I*_Amil_ that became apparent after ~45 min and developed throughout the remainder of the experimental period. Analysis of the data recorded after 3 h revealed a clear increase in the magnitude of *I*_Amil_ (Fig. 5A). These data thus confirm that dexamethasone stimulates electrogenic Na^+^ absorption and further analysis established that the dexamethasone‐induced increase in *J*_Na_ (Δ*J*_Na_) was ~10 mmol cm^−2 ^min^−1^ (Fig. 5B). Figure 5A also includes the data from cyclohexamide‐treated (10 *μ*g ml^−1^, 30 min pre incubation) cells. When applied to hormone‐deprived cells, cyclohexamide caused a slowly developing decline in the magnitude of *I*_Amil_. *R*_t_ consistently remained above 1 kΩ∙cm^2^ throughout all such experiments indicating that the cultured epithelial layers retain their integrity, an observation which makes it highly unlikely that the cyclohexamide‐induced fall in *I*_Amil_ reflects a nonspecific toxic action. We therefore conclude that cyclohexamide causes a progressive fall in basal Na^+^ absorption. While such a decline also occurred in dexamethasone‐stimulated cells, the effect was not as marked and analysis of the currents recorded after 3 h revealed a clear increase in the magnitude of *I*_Amil_. While dexamethasone can thus stimulate Na^+^ transport in the presence of cyclohexamide the magnitude of this response was reduced by ~50 %. Figure 5C shows the results of experiments that explored the effects of cyclohexamide upon the response to insulin. The control data confirm that insulin augments *I*_Amil_ and, as anticipated, this response was smaller (Δ*J*_Na_ ~5 mmol cm^−2^ min^−1^) and more rapid than the response to dexamethasone (Fig. 5C). Although a small effect upon basal *I*_Amil_ was evident, cyclohexamide did not alter the electrometric response to insulin (Fig 5C and D).

## Discussion

### The response to dexamethasone

Although SGK1 is encoded by a glucocorticoid‐inducible gene, SGK1 must be phosphorylated at Ser^422^ and Thr^256^ in order to become catalytically active and the phosphorylation of these physiologically important residues is catalyzed by TORC2 and PDK1, respectively. Moreover, since the activities of TORC2/PDK1 are dependent upon PI3K (Kobayashi and Cohen 1999; Park et al. 1999; García‐Martínez and Alessi 2008; Lu et al. 2010; Mansley and Wilson 2010a), pharmacological inhibition of PI3K disrupts the hormonal control of ENaC by depriving the cell of SGK1 activity (Record et al. 1998; Blazer‐Yost et al. 1999; Păunescu et al. 2000; Blazer‐Yost and Nofziger 2005; Inglis et al. 2009; Mansley and Wilson 2010b). PI3K is therefore central to the hormonal control of epithelial Na^+^ transport and studies of several cell types, including epithelial cells from the ASDN, suggest that steroid hormones can activate this regulatory kinase by inducing transactivation of the IGF‐1 receptor (Helms et al. 2005; Holzman et al. 2007; Cascella et al. 2010; Huang et al. 2012). These findings suggest that steroids might increase cellular SGK1 activity via a dual mechanism involving increased transcription of the *sgk1* gene and activation of PI3K. Our data show that the dexamethasone‐induced increase in SGK1 activity is accompanied by increased abundance of SGK1 protein and mRNA, and in contrast to earlier work (Helms et al. 2005; Holzman et al. 2007; Cascella et al. 2010; Huang et al. 2012) we find that dexamethasone has no effect upon cellular PI3K activity. While we cannot exclude the possibility that dexamethasone might be able to activate PI3K under certain conditions, it is important to stress that our analyses of extracted protein and our electrometric assays of ENaC activity were undertaken using cells grown under identical conditions. The present data therefore show clearly that dexamethasone can increase cellular SGK1 activity, and regulate Na^+^ transport, by evoking SGK1 expression in the absence of any increase in PI3K activity. While this conclusion was initially confusing since a substantial body of earlier work shows that PI3K is central to the control of Na^+^ transport (Record et al. 1998; Blazer‐Yost et al. 1999; Kobayashi and Cohen 1999; Park et al. 1999; Păunescu et al. 2000; Blazer‐Yost and Nofziger 2005; García‐Martínez and Alessi 2008; Inglis et al. 2009; Mansley and Wilson 2010b), the simplest explanation of these findings is that the basal level of PI3K activity is sufficient to ensure phosphorylation of SGK1. Under these conditions, changes to the abundance of SGK1 abundance would be transduced into changes in SGK1 activity.

### The response to insulin

Previous studies of mpkCCD_cl4_ cells (Gonzalez‐Rodriguez et al. 2007) indicated that the magnitude of the Na^+^ current induced by aldosterone and/or insulin‐like growth factor‐1 (IGF‐1), a peptide hormone that activates the same signaling pathway as insulin (Cohen 2006), was proportional to the abundance of SGK1 protein. While aldosterone did evoke SGK1 synthesis more effectively than IGF‐1, it was therefore concluded that both hormone classes contributed to the control of SGK1 transcription (Gonzalez‐Rodriguez et al. 2007). This conclusion was, however, primarily based upon assays of SGK1 abundance (Gonzalez‐Rodriguez et al. 2007) and an important aspect of this study is that we also monitored cellular SGK1 activity (Murray et al. 2004; Inglis et al. 2009). Our studies clearly show that insulin‐induced Na^+^ transport was accompanied by an increase in cellular SGK1 activity (see also Wang et al. 2001; Faletti et al. 2002; Gonzalez‐Rodriguez et al. 2007). Although some authors have suggested that insulin might regulate Na^+^ transport via a mechanism dependent upon PKB rather than SGK1 (Lee et al. 2007; Diakov et al. 2010), earlier studies of the cells used in the present experiments have shown that GSK650394, a small molecule inhibitor of SGK1, suppresses insulin‐evoked Na^+^ transport without inactivating PKB (Mansley and Wilson 2010b), a protein kinase that is closely related to SGK1. Moreover, studies of SGK1^‐/‐^ mice clearly showed that insulin‐evoked Na^+^ retention is dependent upon SGK1 (Huang et al. 2006; Lang et al. 2006) and we therefore conclude that the present increase in cellular SGK1 activity is of central importance to the stimulation of Na^+^ transport. However, our data also show that this increase in cellular SGK1 activity occurred with no change to the abundance of SGK1 protein or mRNA and so our data, in contrast to the results of previous studies (Gonzalez‐Rodriguez et al. 2007), indicate that insulin activates SGK1 via a mechanism, that is, independent of increased *sgk1* expression. Although IGF‐1 receptors and insulin receptors seem to activate the same signaling pathway (Cohen 2006), it is possible that small differences between the properties of these receptors might underlie the discrepancy with earlier work (Gonzalez‐Rodriguez et al. 2007). However, the important point to emerge from our data is that they show that it is possible for insulin to control SGK1 activity, and Na^+^ transport, via a mechanism that does not involve *sgk1* gene transcription. Although the responses to insulin occurred independently of increased *sgk1* gene transcription, this hormone did activate PI3K and insulin would thus increase cellular SGK1 activity by promoting phosphorylation of the already present pool of SGK1. This conclusion accords well with data from earlier studies of amphibian A6 renal epithelial cells (Wang et al. 2001; Faletti et al. 2002) and with recent studies in murine type II alveolar cells (Zhu et al. 2012; He et al. 2015).

### Effects of cyclohexamide

Since the responses to dexamethasone and insulin seem to be differentiated by their requirement for synthesis of the SGK1 protein, we explored the effects of cyclohexamide, a substance known to suppress protein synthesis in mammalian cells, upon the responses to these hormones. The first such studies showed that cyclohexamide reduced cellular SGK1 activity in hormone‐deprived cells and this was anticipated since the cytoplasmic half‐life of SGK1 is only ~30 min (Kobayashi and Cohen 1999; Brickley et al. 2002; Engelsberg et al. 2006). Inhibition of protein synthesis would therefore deplete cellular SGK1 activity over 2–3 h. Moreover, although it had no effect upon the abundance of SGK1 mRNA, cyclohexamide blocked the effects of dexamethasone upon SGK1 activity and abundance and this finding is consistent with the hypothesis that dexamethasone acts by evoking *sgk1* gene expression. Electrometric studies showed that cyclohexamide caused a slowly developing reduction in the rate of electrogenic Na^+^ absorption in hormone‐deprived cells. Although this accords well with the observed fall in cellular SGK1 activity, this progressive decline in basal *I*_Amil_ complicated the analysis of these data. However, since all experiments were undertaken using strictly paired protocols, we were able to compare the currents recorded from hormone‐deprived and dexamethasone‐treated cells. This analysis showed that cyclohexamide caused ~ 50% inhibition of the electrometric response to dexamethasone and, since cyclohexamide had no effect upon the electrometric response to insulin, these data support the idea that dexamethasone and insulin display a differential requirement for SGK1 synthesis.

We were, however, surprised to observe that cyclohexamide also abolished the insulin‐induced activation of SGK1. Our analyses of extracted proteins provided a physiological basis for this effect by showing that cyclohexamide also reduced cellular PI3K activity and abolished the effects of insulin upon this regulatory kinase. Since it is abundantly clear that the catalytic activity of SGK1 is critically dependent upon SGK1, these observations raise the possibility that at least some of the effects of cyclohexamide upon SGK1 which we now report may be secondary to loss of PI3K activity. However, the fact that cyclohexamide can block the effects of insulin upon cellular SGK1 activity is difficult to reconcile with the fact that this inhibitor of protein synthesis had no discernible effect upon the electrometric response to this hormone. While this observation clearly suggests that the electrometric response to insulin may involve mediators other that PI3K/SGK1, this possibility is very difficult to reconcile with the substantial body of data which identify PI3K as the intracellular effector that mediates responses to insulin (see review Cohen 2006). While it is possible that the stimulation of Na^+^ transport may be secondary to increased activity of K^+^ channels, which would increase the driving force for Na^+^ efflux via ENaC by hyperpolarizing the membrane potential, this does appear unlikely since insulin appears to control K^+^ channel activity via a mechanism that is dependent upon PI3K (Zaika et al. 2016). Moreover, although insulin does appear to evoke signaling through extracellular signal‐regulated kinases (ERK1/2) via a mechanism that is independent of PI3K, this pathway generally appears to mediate a reduction in ENaC activity in most epithelial cell types (see review Baines 2013). However, it is important to stress that the protocol used in these experiments allowed us to quantify Na^+^ transport continuously, whereas changes in SGK1 abundance/activity could only be quantified at the end of each experiment after the cells had been lysed. Since the effects of cyclohexamide will develop progressively, effects of SGK1 activity/abundance that occur during the early part of each experiment may not be detected. Moreover, this finding also highlights the fact that global inhibition of protein synthesis will have complex effects upon almost all aspects of cellular physiology. Although these experiments provided some support for the hypothesis that dexamethasone and insulin have different effects on protein synthesis, they also provided inconsistent and confusing results which suggest that it may be inappropriate to use such a drug with such a broad mode of action to explore such a defined aspect of cell physiology.

## Conclusions

Earlier work identified SGK1 as an important part of the mechanism that underlies the hormonal control of ENaC‐mediated Na^+^ retention and showed that this kinase forms a point of convergence between the signaling pathways activated by insulin and by steroids (Wang et al. 2001; Gonzalez‐Rodriguez et al. 2007). While our data are consistent with earlier work which shows that glucocorticoids act by inducing *sgk1* gene expression (Wang et al. 2001; Faletti et al. 2002; Itani et al. 2002; Gonzalez‐Rodriguez et al. 2007), we also establish that dexamethasone has no effect upon cellular PI3K activity. Insulin, on the other hand, activates SGK1 via a mechanism that involves an increase in PI3K activity but no change to *sgk1* gene expression. Although both hormones activate SGK1 and regulate Na^+^ retention, these responses involve clearly distinct physiological mechanisms.

## Conflict of Interest

None declared.

## References

[phy212792-bib-0001] Alessi, D. R. , M. Andjelkovic , B. Caudwell , P. Cron , N. Morrice , P. Cohen , et al. 1996 Mechanism of activation of protein kinase B by insulin and IGF‐1. EMBO J. 15:6541–6551. 8978681 PMC452479

[phy212792-bib-0002] Bailey, M. A. , J. M. Paterson , P. W. F. Hadoke , N. Wrobel , C. O. C. Bellamy , D. G. Brownstein , et al. 2008 A switch in the mechanism of hypertension in the syndrome of apparent mineralocorticoid excess. J. Am. Soc. Nephrol. 19:47–58. 18032795 10.1681/ASN.2007040401 PMC2391031

[phy212792-bib-0003] Bailey, M. A. , J. J. Mullins , and C. J. Kenyon . 2009 Mineralocorticoid and glucocorticoid receptors stimulate epithelial sodium channel activity in a mouse model of cushing syndrome. Hypertension 54:890–896. 19635986 10.1161/HYPERTENSIONAHA.109.134973

[phy212792-bib-0004] Baines, D. L. 2013 Kinases as targets for ENaC regulation. Curr. Mol. Pharmacol. 6:50–64. 23547935 10.2174/18744672112059990028

[phy212792-bib-0005] Bayascas, J. R. , and D. R. Alessi . 2005 Regulation of Akt/PKB Ser^473^ phosphorylation. Mol. Cell 18:143–145. 15837416 10.1016/j.molcel.2005.03.020

[phy212792-bib-0006] Bens, M. , V. Vallet , F. Cluzeaud , L. Pascual‐Letallec , A. Kahn , M. E. Rafestin‐Oblin , et al. 1999 Corticosteroid‐dependent sodium transport in a novel immortalized mouse collecting duct principal cell line. J. Am. Soc. Nephrol. 10:923–934. 10232677 10.1681/ASN.V105923

[phy212792-bib-0007] Blazer‐Yost, B. L. , and C. Nofziger . 2005 The role of the phosphoinositide pathway in hormonal regulation of the epithelial sodium channel. Adv. Exp. Med. Biol. 559:359–368. 18727255 10.1007/0-387-23752-6_33

[phy212792-bib-0008] Blazer‐Yost, B. L. , X. Liu , and S. I. Helman . 1998 Hormonal regulation of ENaCs: insulin and aldosterone. Am. J. Physiol. – Cell Physiol. 274:C1373–C1379. 10.1152/ajpcell.1998.274.5.C1373 9612225

[phy212792-bib-0009] Blazer‐Yost, B. L. , T. G. Păunescu , S. I. Helman , K. D. Lee , and C. J. Vlahos . 1999 Phosphoinositide 3‐kinase is required for aldosterone‐regulated sodium reabsorption. Am. J. Physiol. – Cell Physiol. 277:C531–C536. 10.1152/ajpcell.1999.277.3.C531 10484339

[phy212792-bib-0010] Brickley, D. R. , C. A. Mikosz , C. R. Hagan , and S. D. Conzen . 2002 Ubiquitin modification of serum and glucocorticoid‐induced protein kinase‐1 (SGK‐1). J. Biol. Chem. 277:43064–43070. 12218062 10.1074/jbc.M207604200

[phy212792-bib-0011] Cascella, T. , Y. Radhakrishnan , L. A. Maile , W. H. Busby , K. Gollahon , A. Colao , et al. 2010 Aldosterone enhances IGF‐I‐mediated signaling and biological function in vascular smooth muscle cells. Endocrinology 151:5851–5864. 20881255 10.1210/en.2010-0350 PMC2999491

[phy212792-bib-0012] Cohen, P. 2006 The twentieth century struggle to decipher insulin signalling. Nat. Rev. Mol. Cell Biol. 7:867–873. 17057754 10.1038/nrm2043

[phy212792-bib-0013] De La Rosa, A. D. , P. Zhang , A. Náray‐Fejes‐Tóth , G. Fejes‐Tóth , and C. M. Canessa . 1999 The serum and glucocorticoid kinase (sgk) increases the abundance of epithelial sodium channels in the plasma membrane of Xenopus oocytes. J. Biol. Chem. 274:37834–37839. 10608847 10.1074/jbc.274.53.37834

[phy212792-bib-0014] De La Rosa, A. D. , H. Li , and C. M. Canessa . 2002 Effects of aldosterone on biosynthesis, traffic, and functional expression of epithelial sodium channels in A6 cells. J. Gen. Physiol. 119:427–442. 11981022 10.1085/jgp.20028559 PMC2233818

[phy212792-bib-0015] Diakov, A. , V. Nesterov , M. Mokrushina , R. Rauh , and C. Korbmacher . 2010 Protein Kinase B Alpha (PKB alpha) stimulates the Epithelial Sodium Channel (ENaC) heterologously expressed in xenopus laevis oocytes by two distinct mechanisms. Cell. Physiol. Biochem. 26:913–924. 21220922 10.1159/000324000

[phy212792-bib-0016] Engelsberg, A. , F. Kobelt , and D. Kuhl . 2006 The N‐terminus of the serum‐ and glucocorticoid‐inducible kinase Sgk1 specifies mitochondrial localization and rapid turnover. Biochem. J. 399:69–76. 16776652 10.1042/BJ20060386 PMC1570167

[phy212792-bib-0017] Faletti, C. J. , N. Perrotti , S. I. Taylor , and B. L. Blazer‐Yost . 2002 sgk: an essential convergence point for peptide and steroid hormone regulation of ENaC‐mediated Na^+^ transport. Am. J. Physiol. – Cell Physiol. 282:C494–C500. 11832334 10.1152/ajpcell.00408.2001

[phy212792-bib-0018] García‐Martínez, J. M. , and D. R. Alessi . 2008 mTOR complex 2 (mTORC2) controls hydrophobic motif phosphorylation and activation of serum‐ and glucocorticoid‐induced protein kinase 1 (SGK1). Biochem. J. 416:375–385. 18925875 10.1042/BJ20081668

[phy212792-bib-0019] Gonzalez‐Rodriguez, E. , H. P. Gaeggeler , and B. C. Rossier . 2007 IGF‐1 vs insulin: respective roles in modulating sodium transport via the PI‐3 kinase/Sgk1 pathway in a cortical collecting duct cell line. Kidney Int. 71:116–125. 17164836 10.1038/sj.ki.5002018

[phy212792-bib-0020] Guan, Y. , C. Hao , D. R. Cha , R. Rao , W. Lu , D. E. Kohan , et al. 2005 Thiazolidinediones expand body fluid volume through PPAR*γ* stimulation of ENaC‐mediated renal salt absorption. Nat. Med. 11:861–866. 16007095 10.1038/nm1278

[phy212792-bib-0021] He, J. , D. Qi , D.‐X. Wang , W. Deng , Y. Ye , L.‐H. Feng , et al. 2015 Insulin upregulates the expression of epithelial sodium channel in vitro and in a mouse model of acute lung injury: role of mTORC2/SGK1 pathway. Exp. Cell Res. 331:164–175. 25265063 10.1016/j.yexcr.2014.09.024

[phy212792-bib-0022] Helms, M. N. , L. Liu , Y. Y. Liang , O. Al‐Khalili , A. Vandewalle , S. Saxena , et al. 2005 Phosphatidylinositol 3,4,5‐trisphosphate mediates aldosterone stimulation of epithelial sodium channel (ENγaC) and interacts with Î³‐ENaC. J. Biol. Chem. 280:40885–40891. 16204229 10.1074/jbc.M509646200

[phy212792-bib-0023] Holzman, J. L. , L. Liu , B. J. Duke , A. E. Kemendy , and D. C. Eaton . 2007 Transactivation of the IGF‐1R by aldosterone. Am. J. Physiol. ‐ Renal Physiol. 292:F1219–F1228. 17190911 10.1152/ajprenal.00214.2006

[phy212792-bib-0024] Huang, D. Y. , K. M. Boini , B. Friedrich , M. Metzger , L. Just , H. Osswald , et al. 2006 Blunted hypertensive effect of combined fructose and high‐salt diet in gene‐targeted mice lacking functional serum‐ and glucocorticoid‐inducible kinase SGK1. Am. J. Physiol. Regul. Integr. Comp. Physiol. 290:R935–R944. 16284089 10.1152/ajpregu.00382.2005

[phy212792-bib-0025] Huang, L. L. , D. J. Nikolic‐Paterson , F. Y. Ma , and G. H. Tesch . 2012 Aldosterone induces kidney fibroblast proliferation via activation of growth factor receptors and PI3K/MAPK signalling. Nephron Exp. Nephrol. 120:e115–e122. 22814207 10.1159/000339500

[phy212792-bib-0026] Hunter, R.W. , J. R. Ivy , and M. A. Bailey . 2014 Glucocorticoids and renal sodium transport: implications for hypertension and salt‐sensitivity. J. Physiol. 592:1731–1744. 24535442 10.1113/jphysiol.2013.267609 PMC4001748

[phy212792-bib-0027] Inglis, S. K. , M. Gallacher , S. G. Brown , N. McTavish , J. Getty , E. M. Husband , et al. 2009 SGK1 activity in Na^+^ absorbing airway epithelial cells monitored by assaying NDRG1‐Thr^346/356/366^ phosphorylation. Pflugers Arch. 457:1287–1301. 18787837 10.1007/s00424-008-0587-1

[phy212792-bib-0028] Itani, O. A. , K. Z. Liu , K. L. Cornish , J. R. Campbell , and C. P. Thomas . 2002 Glucocorticoids stimulate human sgk1 gene expression by activation of a GRE in its 5’‐flanking region. Am. J. Physiol. – Endocrinol Metabol. 283:E971–E979. 10.1152/ajpendo.00021.2002 12376324

[phy212792-bib-0029] Kobayashi, T. , and P. Cohen . 1999 Activation of serum‐ and glucocorticoid‐regulated protein kinase by agonists that activate phosphatidylinositide 3‐kinase is mediated by 3‐phosphoinositide‐dependent protein kinase‐1 (PDK1) and PDK2. Biochem. J. 339:319–328. 10191262 PMC1220160

[phy212792-bib-0030] Lang, F. , C. Böhmer , M. Palmada , G. Seebohm , N. Strutz‐Seebohm , and V. Vallon . 2006 (Patho)physiological significance of the serum‐ and glucocorticoid‐ inducible kinase isoforms. Physiol. Rev. 86:1151–1178. 17015487 10.1152/physrev.00050.2005

[phy212792-bib-0031] Lee, I. H. , A. Dinudom , A. Sanchez‐Perez , S. Kumar , and D. I. Cook . 2007 Akt mediates the effect of insulin on epithelial sodium channels by inhibiting Nedd4‐2. J. Biol. Chem. 282:29866–29873. 17715136 10.1074/jbc.M701923200

[phy212792-bib-0032] Loffing, J. , and C. Korbmacher . 2009 Regulated sodium transport in the renal connecting tubule (CNT) via the epithelial sodium channel (ENaC). Pflügers Arch. 458:111–135. 19277701 10.1007/s00424-009-0656-0

[phy212792-bib-0033] Lu, M. , J. Wang , K. T. Jones , H. E. Ives , M. E. Feldman , L. J. Yao , et al. 2010 mTOR complex‐2 activates ENaC by phosphorylating SGK1. J. Am. Soc. Nephrol. 21:811–818. 20338997 10.1681/ASN.2009111168 PMC2865740

[phy212792-bib-0034] Mansley, M. K. , and S. M. Wilson . 2010a Dysregulation of epithelial Na^+^ absorption induced by inhibition of the kinases TORC1 and TORC2. Br. J. Pharmacol. 161:1778–1792. 20735411 10.1111/j.1476-5381.2010.01003.x PMC3010582

[phy212792-bib-0035] Mansley, M. K. , and S. M. Wilson . 2010b Effects of nominally selective inhibitors of the kinases PI3K, SGK1 and PKB on the insulin‐dependent control of epithelial Na^+^ absorption. Br. J. Pharmacol. 161:571–588. 20880397 10.1111/j.1476-5381.2010.00898.x PMC2990156

[phy212792-bib-0036] May, A. , A. Puoti , H. Gaeggeler , J. Horisberger , and B. C. Rossier . 1997 Early effect of aldosterone on the rate of synthesis of the epithelial sodium channel α subunit in A6 renal cells. J. Am. Soc. Nephrol. 8:1813–1822. 9402082 10.1681/ASN.V8121813

[phy212792-bib-0037] McTavish, N. , J. Getty , A. Burchell , and S. M. Wilson . 2009 Glucocorticoids can activate the *α*‐ENaC gene promoter independently of SGK1. Biochem. J. 423:189–197. 19619128 10.1042/BJ20090366 PMC2762689

[phy212792-bib-0038] Murray, J. T. , D. G. Campbell , N. Morrice , G. C. Auld , N. Shpiro , R. Marquez , et al. 2004 Exploitation of KESTREL to identify NDRG family members as physiological substrates for SGK1 and GSK3. Biochem. J. 384:477–488. 15461589 10.1042/BJ20041057 PMC1134133

[phy212792-bib-0039] Murray, J. T. , L. A. Cummings , G. B. Bloomberg , and P. Cohen . 2005 Identification of different specificity requirements between SGK1 and PKB alpha. FEBS Lett. 579:991–994. 15710380 10.1016/j.febslet.2004.12.069

[phy212792-bib-0040] Park, J. , M. L. L. Leong , P. Buse , A. C. Maiyar , G. L. Firestone , and B. A. Hemmings . 1999 Serum and glucocorticoid‐inducible kinase (SGK) is a target of the PI 3‐kinase‐stimulated signaling pathway. EMBO J. 18:3024–3033. 10357815 10.1093/emboj/18.11.3024 PMC1171384

[phy212792-bib-0041] Păunescu, T. G. , B. L. Blazer‐Yost , C. J. Vlahos , and S. I. Helman . 2000 LY‐294002‐inhibitable PI 3‐kinase and regulation of baseline rates of Na^+^ transport in A6 epithelia. Am. J. Physiol. – Cell Physiol. 279:C236–C247. 10898735 10.1152/ajpcell.2000.279.1.C236

[phy212792-bib-0042] Record, R. D. , L. L. Froelich , C. J. Vlahos , and B. L. Blazer‐Yost . 1998 Phosphatidylinositol 3‐kinase activation is required for insulin‐ stimulated sodium transport in A6 cells. Am. J. Physiol. – Endocrinol Metabol. 274:C531–C536. 10.1152/ajpendo.1998.274.4.E611 9575821

[phy212792-bib-0043] Sarbassov, D. D. , D. A. Guertin , S. M. Ali , and D. M. Sabatini . 2005 Phosphorylation and regulation of Akt/PKB by the rictor‐mTOR complex. Science 307:1098–1101. 15718470 10.1126/science.1106148

[phy212792-bib-0044] Sayegh, R. , S. D. Auerbach , X. Li , R. W. Loftus , R. F. Husted , J. B. Stokes , et al. 1999 Glucocorticoid induction of epithelial sodium channel expression in lung and renal epithelia occurs via trans‐activation of a hormone response element in the 5 ‘‐flanking region of the human epithelial sodium channel alpha subunit gene. J. Biol. Chem. 274:12431–12437. 10212217 10.1074/jbc.274.18.12431

[phy212792-bib-0045] Scott, C. L. , D. J. Walker , E. Cwiklinski , C. Tait , A. R. Tee , and S. C. Land . 2010 Control of HIF‐1*α* and vascular signaling in fetal lung involves cross talk between mTORC1 and the FGF‐10/FGFR2b/Spry2 airway branching periodicity clock. Am. J. Physiol. – Lung Cell Mol Physiol. 299:L455–L471. 20622121 10.1152/ajplung.00348.2009 PMC2957420

[phy212792-bib-0046] Wang, J. , P. Barbry , A. C. Maiyar , D. J. Rozansky , A. Bhargava , M. Leong , et al. 2001 SGK integrates insulin and mineralocorticoid regulation of epithelial sodium transport. Am. J. Physiol. – Renal Physiol. 280:F303–F313. 11208606 10.1152/ajprenal.2001.280.2.F303

[phy212792-bib-0047] Wilson, S. M. , M. K. Mansley , J. Getty , E. M. Husband , S. K. Inglis , and M. K. Hansen . 2010 Effects of peroxisome proliferator‐activated receptor *γ* agonists on Na^+^ transport and activity of the kinase SGK1 in epithelial cells from lung and kidney. Br. J. Pharmacol. 159:678–688. 20105179 10.1111/j.1476-5381.2009.00564.x PMC2828031

[phy212792-bib-0048] Zaika, O. , O. Palygin , V. Tomilin , M. Mamenko , A. Staruschenko , and O. Pochynyuk . 2016 Insulin and IGF‐1 activate Kir4.1/5.1 channels in cortical collecting duct principal cells to control basolateral membrane voltage. Am. J. Physiol. – Renal Physiol. 310:F311–F321. 26632606 10.1152/ajprenal.00436.2015 PMC4839479

[phy212792-bib-0049] Zhu, T. , W. Zhang , and D.‐X. Wang . 2012 Insulin up‐regulates epithelial sodium channel in LPS‐induced acute lung injury model in rats by SGK1 activation. Injury 43:1277–1283. 22552040 10.1016/j.injury.2012.04.004

